# CardioGxE, a catalog of gene-environment interactions for cardiometabolic traits

**DOI:** 10.1186/1756-0381-7-21

**Published:** 2014-10-26

**Authors:** Laurence D Parnell, Britt A Blokker, Hassan S Dashti, Paula-Dene Nesbeth, Brittany Elle Cooper, Yiyi Ma, Yu-Chi Lee, Ruixue Hou, Chao-Qiang Lai, Kris Richardson, José M Ordovás

**Affiliations:** 1 JM-USDA Human Nutrition Research Center on Aging at Tufts University, 711 Washington Street, Boston, MA 02111, USA

**Keywords:** Cardiovascular diseases, Diet, Gene-environment interaction, Genetic variants, Phenotypic variance, Physical activity, Type 2 diabetes

## Abstract

**Background:**

Genetic understanding of complex traits has developed immensely over the past decade but remains hampered by incomplete descriptions of contribution to phenotypic variance. Gene-environment (GxE) interactions are one of these contributors and in the guise of diet and physical activity are important modulators of cardiometabolic phenotypes and ensuing diseases.

**Results:**

We mined the scientific literature to collect GxE interactions from 386 publications for blood lipids, glycemic traits, obesity anthropometrics, vascular measures, inflammation and metabolic syndrome, and introduce CardioGxE, a gene-environment interaction resource. We then analyzed the genes and SNPs supporting cardiometabolic GxEs in order to demonstrate utility of GxE SNPs and to discern characteristics of these important genetic variants. We were able to draw many observations from our extensive analysis of GxEs. 1) The CardioGxE SNPs showed little overlap with variants identified by main effect GWAS, indicating the importance of environmental interactions with genetic factors on cardiometabolic traits. 2) These GxE SNPs were enriched in adaptation to climatic and geographical features, with implications on energy homeostasis and response to physical activity. 3) Comparison to gene networks responding to plasma cholesterol-lowering or regression of atherosclerotic plaques showed that GxE genes have a greater role in those responses, particularly through high-energy diets and fat intake, than do GWAS-identified genes for the same traits. Other aspects of the CardioGxE dataset were explored.

**Conclusions:**

Overall, we demonstrate that SNPs supporting cardiometabolic GxE interactions often exhibit transcriptional effects or are under positive selection. Still, not all such SNPs can be assigned potential functional or regulatory roles often because data are lacking in specific cell types or from treatments that approximate the environmental factor of the GxE. With research on metabolic related complex disease risk embarking on genome-wide GxE interaction tests, CardioGxE will be a useful resource.

## Background

Over the last decade, hundreds of genetic loci have been described as contributors to complex traits and human diseases. Yet, often a large proportion of the heritability of many traits remains ill defined. Contributors to phenotypic variance include: inability of small sample sizes to detect variants with small effects, disease markers not in complete linkage disequilibrium (LD) with the causal variant thus underestimating heritability [1], heritability overestimation from family-based populations, rare or “private” mutations [2,3], inherited patterns of epigenetic marks [4], epistasis (gene-gene interactions) [5,6] and gene-environment (GxE) interactions [7,8]. Of these, the GxE has drawn much attention in part because it describes a modifiable relationship between genetic variation and changes in phenotype, one by which an individual can take action with potential health benefits.

The cell and the organism as a whole are consistently challenged to maintain homeostasis in the face of a wide array of stimuli or perturbations, both health-promoting and disease-causing. To accomplish homeostasis, adjustments to molecular parameters must be enacted that correspond to the stimulatory challenge, which typically includes altered protein function or gene expression. This all amounts to continual changes to the phenotypes of the cell or organism and it is the timeliness and efficiency of these phenotypic adjustments that determine health and healthy aging. This process can be termed phenotypic flexibility, a phenomenon which is a central concept of the gene-environment interaction [9]. A gene-environment interaction refers to modification by an environmental factor of the effect of a genetic variant on a phenotypic trait [10]. Environmental factors can include diet, dietary components such as saturated fatty acids, physical activity, sedentary behavior, alcohol, or sleep, among many others. Such GxE interactions can serve to modulate the adverse effects of a risk allele, or can exacerbate the genotype-phenotype relationship and increase risk. Additionally, environmental stimuli, acting over hundreds of generations, can promote adaptation that is observed in current populations as affecting disease risk [11]. Importantly, a complete catalog of GxEs for a given phenotype will provide the means whereby an individual can adjust exposure to a particular environmental factor involved in GxE interactions for the benefit of lessening disease risk according to a fixed genotype.

The genetic basis of transcription rates, particularly as responses to stimuli, and transcription differences between individuals is now widely recognized as commonplace, with important consequences on disease outcomes. Expression quantitative trait loci (eQTL) are currently seen as one important source linking discovery of disease genes to functional mechanisms that are the basis of complex traits [12]. Similarly, loci supporting GxE interactions contribute to variance of complex traits in a manner involving an environmental factor or stimulus and thus likely also represent response eQTL. In addition, genetic variation in cardiometabolic traits results in part from adaption to local environments [13]. Thus, genetic variants that have been subject to positive selection, can interact with environmental factors, such as climate, diet, and lifestyle, leading to increased risk in cardiometabolic diseases [13].

Our 2011 report cataloged 554 GxE interactions, 377 of which contained common traits and environmental factors, that reached statistical significance and were pertinent to nutrition, cardiovascular diseases, blood lipids and type 2 diabetes mined from 184 scientific reports [14]. We inventoried more GxEs for HDL-cholesterol as phenotype and physical activity as modifying environmental factor than any other terms in the GxE equation. Overall, obesity anthropometrics was also a leading phenotype with body mass index (BMI) predominating the significant obesity GxEs. As a result of increased GxE reports, the objectives of the current study were to update our 2011 report and show the broad utility of GxE interactions to population genetics and human disease by comparing to other biomedical genomics data.

## Methods

The description of literature mining and building this dataset has been described [14]. Briefly, articles available before September, 2013 were queried at PubMed or http://www.quertle.info with search terms including genetic variation (e.g., SNP, variant, polymorphism), “interaction,” or an environmental factor (e.g., diet, physical activity or exercise, alcohol, sleep, tobacco/cigarette) and, after reading and manual parsing of the data, were incorporated into the update presented here. Specifically, data fields captured included SNPs tested for GxE interactions, the assigned gene for the SNP, common aliases of the SNP, risk allele, phenotype, modifying environmental factor, population ethnicity/origin and PubMed identifier. We excluded all reports on children and adolescents, and any GxE studies examining non-alcoholic fatty liver disease and other phenotypes that are peripherally affiliated with cardiometabolic dysfunction, including atrial fibrillation, cardiomyopathies and response to lipid-lowering, glucose-homeostasis and other medications.

To demonstrate the utility of GxE SNPs within the CardioGxE dataset and interactions they represent, and to offer insight into potential mechanisms of function, we performed a series of comparisons to other biomedical genomics data. These comparisons to test for enrichment included roles in main-effect associations to disease phenotypes, transcriptional control (either via allele-specific expression, microRNA-mRNA interaction or epigenetics), adaptation, and in maintaining metabolic homeostasis in a set of pertinent tissues and cell types. To initiate these analyses, we created two separate SNP datasets based on LD: one for GxE SNPs and another from genome-wide association studies (GWAS) SNPs for the same cardiometabolic traits but not including any SNPs for which there is GxE evidence. Genomic coordinates (dbSNP138) for the region spanning 300 kb and centered on each SNP were determined. A bash shell script was written to retrieve iteratively all 1000 Genomes Project SNP data (accessed 04/10/2014) within this region from the CEU population using tabix and vcftools [15], pipe these data into Haploview for LD analysis using a *r*^2^ ≥ 0.80, and return all variants contained in the LD block of the input SNP [16]. These SNPs were used for further analysis. Significance of enrichment in a comparison between two datasets was performed by two sample *z*-test.

Two measures of positive selection signals for GxE SNPs, integrated haplotype score (iHS) [17] and global Fst, were acquired from data extracted from the 1000 Genome Selection Browser 1.0 [18]. SNPs with |iHS| ≥ 2.0 [17], or Fst ≥0.5 [19] were considered as subject to positive selection [17]. For the control, positive selection signals of a matched set of SNPs of significant main effects, but without known GxE interaction, were also obtained from the 1000 Genome Selection Browser 1.0. To determine the enrichment of positive selection variants in GxE interactions, the *Z*-score test was conducted.

To determine if a GxE SNP or one in LD had evidence of *cis* or *trans* eQTL data, we collected significant hits from 5 published eQTL experiments [20-24]. A Perl script was written to search GxE SNPs against each list of significant eQTL hits.

On the basis of our earlier microRNA (miR) target SNP database [25], we further collected human SNPs that are potentially involved in miR targeting regulation by using miR target prediction algorithms TargetScan [26], TargetScanS, miRanda [27], microRNA.org [28], PITA [29], PicTar [30], mirsnpscore [31] and dbSMR [32]. Targets were downloaded with genome coordinates and mapped to genomic positions according to GRCh37/hg19 using the LiftOver tool from the UCSC Genome Browser and supplemented with any dbSNP137 SNPs located in predicted target sites. SNPs also were collected from published miR SNP databases: PolymiRTS [33], PolymiRTS 2.0 [34], PolymiRTS 3.0 [35], Patrocles [36], PupaSuite 3.1 [37], miRdsnp [38], miRNASNP [39], MirSNP [40] miRcode [41] and other literature resources, including predicted and experimentally validated sites. For SNPs located in miR genes, we used the UCSC Genome Browser tract wgRna_sno/miRNA and limited results to miR precursor forms then by searches for any SNPs positioned within gene regions. For genetic variants affecting miR processing machinery, SNPs were identified that mapped within genes encoding these enzymes.

## Results and discussion

### Cardiometabolic GxE interaction catalog

All GxE interaction tests for cardiometabolic traits from 386 published scientific reports identified by literature mining are presented in Additional file 1. We include tests passing the threshold for statistical significance as reported by the study authors, generally *p* <0.05, plus those tests that are not significant. The CardioGxE catalog is composed of 1187 significant GxEs (in 189 genes) and 13770 with no significant interaction observed. By far, most reports examined populations of European ancestry. Of 1187 significant GxEs, 1013 (85.2%) involve the typically measured lifestyle choices or environmental factors of physical activity or inactivity, smoking, alcohol consumption and diet. Dietary measures include macronutrient intakes, either as daily amounts or as percent of total energy, of carbohydrates, both simple and complex; protein; and fat, sub-divided into total fat, saturated fatty acid (SFA), mono-unsaturated fatty acid (MUFA), and poly-unsaturated fatty acid (PUFA), with the latter further categorized as N-3 or N-6, omega-3 or omega-6, respectively. Of 1187 significant GxEs, 992 (83.6%) include the commonly measured phenotypes of blood lipids (HDL-cholesterol, LDL-cholesterol, VLDL-cholesterol, total cholesterol, triglyceride), glycemic traits (type 2 diabetes status, plasma glucose and insulin, HOMA-IR, beta cell function as HOMA-BC), obesity anthropometrics (BMI/obesity, adiposity, body weight, waist circumference, waist-to-hip ratio), vascular measures (diastolic and systolic blood pressure), inflammation (C-reactive protein or CRP), and metabolic syndrome, or changes in these values in response to an intervention, typically dietary.

We then trimmed the data to those significant GxEs that contain both common phenotypes and environmental factors producing a list of 654 different significant cardiometabolic GxEs. These GxEs are different in terms of any data parameter including population, or the direction or threshold of the environmental term constituting the GxE interaction. This dataset, although smaller than the 1187 total GxEs mined from the literature, allows for much more direct comparisons to other biomedical and genomics datasets. In our 2011 report, we described 554 different GxE interactions from 184 publications [14]. In that dataset, we cataloged 377 GxEs containing common phenotypes and common environmental factors. Thus, while we have observed growth in GxEs for cardiometabolic traits over the past three years, there also have been a few large-scale or genome-wide studies, which have produced a substantial number of interactions not reaching significance, as well as greater diversity in both the phenotypes and environmental terms analyzed.

### GxE SNPs involved in genetic-based diseases and GWAS

The National Human Genome Research Institute (NHGRI) maintains a Clinical Genomic Database [42], a manually curated database of conditions with known genetic causes [43]. These data can be queried to obtain genes implicated in certain medical conditions with regard to the clinical utility of genetic diagnosis. We conducted a query on 22 May 2013 for the term “cardiovascular”, which returned 486 different genes, of which 24 have evidence for GxE interactions for cardiometabolic traits. The corollary of this finding is only 24 of 189 (12.7%) cardiometabolic GxE genes are present in the clinical genomic dataset, yet these genes are linked to phenotypes pertinent to cardiovascular diseases. Because this observation is general and without regard to specific phenotypes, we sought to look more deeply at the occurrence of genes shared between the CardioGxE catalog and other datasets of gene-phenotype relationships.

GWAS have been powerful interrogators of the genome, identifying genetic sources of phenotypic variance and disease risk. However, the contribution to phenotype variance that could be explained solely by main effect associations for many cardiometabolic traits was quite small [44]. We reasoned that GxE interactions are important contributors to phenotypic variance. Thus, it would be useful to determine the extent to which sets of genes affiliated with certain cardiometabolic traits also show GxE interactions, as well as how often genes supporting GxE interactions for a given trait have no other evidence linking the gene to that trait. We mined four gene and genetic association databases for genes assigned to four different cardiometabolic traits: blood pressure, HOMA-IR, total cholesterol and LDL-cholesterol. These databases were NCBI Gene, the NHGRI GWAS Catalog [45], the PheGenI phenotype-genotype integrator [46], and a recent comprehensive review of coronary artery disease risk factors [47]. That review lists 326 different genes involved in CAD susceptibility or a series of risk factors ranging from blood lipids to glucometabolic traits and C-reactive protein [47]. None of these four databases contained the same number of genes assigned to a given trait, underscoring the fact that all relationships between gene and phenotype are not comprehensively cataloged in one place. For each phenotype, we observed very few genes shared by our GxE catalog with any of the four gene/genetic association data sources, ranging from a minimum of no genes shared to a maximum of 20% of genes (15 of 75 genes) assigned in the example of LDL-C in NCBI Gene (data not shown).

In order to compare GxE SNPs to SNPs supporting main effect associations, we first compiled a list of SNPs in high LD with the lead GxE SNP. This was done with data from the 1000 Genomes Project in the CEU population with an *r*^2^ threshold set to 0.80 yielding a set of 3381 GxE SNPs. We then compared these GxE SNPs to SNPs supporting main effect associations to cardiometabolic phenotypes in two important resources. Of 759 SNPs with associations to cardiometabolic phenotypes in the GWAS catalog [45], only 36 (4.7%) show evidence of GxE interactions. In addition, of the 3381 GxE SNPs, only 112 (3.3%), representing 146 unique SNP-phenotype pairs, show an association to a cardiometabolic trait as mined from PheGenI [46]. Furthermore, of these 146 SNP-phenotype pairs, only 37, or 25.3% support a GxE interaction for the same or very similar phenotype. Taken together, these observations underscore the incomplete description of contribution to phenotypic variance by main effect associations, and strengthen the importance of GxE interactions as contributors to that variance. This then implies that genetic contributors alone are insufficient diagnostic tools for assessing disease risk, but those calculations also must include at least the GxE term.

### Genetics – GxE and epistasis connections

Epistasis also has been offered as a contributor to the observed variance in disease phenotypes [5,6]. Some groups have undertaken a knowledge-driven approach, using shared relationships from protein-protein interaction data or pathway assignment, to identify potential gene-gene or epistatic interactions [48,49]. In a similar vein, we hypothesized that epistatic alleles could operate via shared mechanistic linkages and that these could then be observed as coordinate pairs of identical GxE interactions. To test this, we collected epistatic relationships for common cardiometabolic traits from the literature and examined those SNP-phenotype relationships in our GxE catalog.

Of eleven significant gene-gene interaction models discovered in a cohort in which epistasis was examined as a source for phenotypic variance for HDL-C [48], only two epistasis pairs were tested for GxE interactions for the same HDL-C trait. One, our catalog lists *ABCA1* and *LPL* markers as each having GxE interactions for HDL-C, but always with environmental factors not shared with the other gene. Two, a knowledge-driven screen of GWAS data reported an interaction between *LIPC* and *HMGCR* for HDL-C [49], but no GxE interactions for *HMGCR* are cataloged here. Additional literature mining revealed several gene-gene interactions acting on cardiometabolic traits. We identified just five examples for which the genes containing the epistasis relationship also participate in GxE interactions for the same phenotype and environmental factor. These include *LEP*x*LEPR* on obesity [50] and a change in BMI-low-calorie diet GxE as well as a BMI-PUFA N-6 linoleic acid GxE; *ADRB2*x*ADRB3* on BMI [51] and a BMI-physical activity GxE; *APOE*x*CETP* on HDL-C [52] and GxE interactions for alcohol, fat intake, physical activity or SFA intake; *CETP*x*LIPC* on HDL-C [53] and GxE interactions with physical activity, percent energy from animal fat, and intakes of fat, MUFA and SFA; and *PPARA*x*PPARG* on small dense LDL [54] and a LDL particle size-SFA intake GxE. Although examination of our GxE catalog shows that the published epistasis gene pairs often are not tested for the same phenotype-environmental factor combination, a number have been tested but few exhibit shared GxE interactions. This may indicate that the pools of genetic factors contributing to phenotypic variance via epistasis and GxE interactions are rather distinct. Comparing to such a small epistasis dataset, however, is insufficient and thus it remains an open question as to how often epistasis genes will share an environmental interaction and reveal any mechanisms of action.

### GxE variants under positive selection

Comparisons of risk allele frequencies across diverse populations have established appreciable directional differentiation for blood lipid and T2DM risk allele frequencies [55,56]. The decreasing frequencies of some T2DM risk alleles seen along an eastward arc from Africa to eastern Asia supplement disparities in predicted genetic risk, such that a portion of T2DM genetic risk is consistently elevated for individuals in African populations and lower in Asian populations [56], but this is somewhat controversial [57]. Accordingly, and considering that geography and climate strongly influence available foodstuffs, seasonally directed energy expenditures and other nutrition-centric human activity [13], we sought to identify those GxE SNPs that show evidence for positive selection.

Three resources were used to investigate relationships between cardiometabolic GxEs and adaptation to climate and geography. First, two genome-wide studies have examined associations between genetic variants and climatic and geographical characteristics, including latitude, seasonality, precipitation, solar radiation and temperature [58,59]. Second, a collection of genes was identified as under selection in different human populations with roles in cultural practices, often with rationales pertinent to agriculture, diet and societal behaviors [60]. Third, a number of other studies have assessed adaptation at candidate loci for specific phenotypes and we chose to examine those germane to cardiometabolic traits. From these reports, we found that 25 of 189 different genes supporting cardiometabolic GxE interactions show adaptation to climatic and geographical characteristics (Table 1). Just 23 of 453 loci participating in main effect associations for these cardiometabolic traits, as mined from the GWAS catalog [45], show adaptation to climate and geography features, indicating significant enrichment in the GxE dataset (*p* <0.001, two sample *z*-test).

**Table 1:** GxE genes and SNPs under selection for climatic and geographic characteristics

**Gene/SNP**	**Climate/Geography factor**	**Cardiometabolic GxE, phenotype-environmental factor**
*ACE*-rs7214530	Winter PC1 [58]	SBP-physical activity; *LC*
*ADAMTS9*	Hypoxia - high-altitude Amharans (Ethiopia) [61]	*LC*
*ADH1C*	Digestion of milk and dairy products; metabolism of carbohydrates, starch, proteins, lipids and phosphates; alcohol metabolism [60]	BMI-alcohol consumption; waist circumference-alcohol consumption
*ADRA2B*-rs7604842	Summer PC1 [58]	T2DM-physical activity; *LC*
*ANGPTL4*	Hypoxia - high-altitude Tibetans [62]	HDL-C-carbohydrate, percent energy from
*APOA4* - rs5110	Nutritional advantage to those with high milk consumption [63]	Varirous GxEs: blood lipids-dietary fatty acid intake
*CD36*	Dispersal and subsequent exposure to novel climates; summer PC1; fat consumption [58,60,64]	*LC*
*CETP*	Dispersal and subsequent exposure to novel climates; energy metabolism and hot or cold tolerance; summer PC2 [58,60]	Cholesterol, total-fat, total intake; Δcholesterol, total-SFA, percent energy from; **HDL-C-diet (3)**; **HDL-C-physical activity (4)**; **HDL-C-alcohol consumption (2)**; HDL-C-fat, total, percent energy from; HDL-C-MUFA, percent energy from; HDL-C-SFA, percent energy from; triglyceride-alcohol consumption; *LC*
*CLOCK*-rs1979605	Summer PC2 [58]	BMI-physical activity; glucose, fasting-MUFA, percent energy from; insulin resistance (HOMA-IR)-MUFA, percent energy from; waist circumference-SFA, percent energy from
*CYP1A2*	Domestication of plants [60]	myocardial infarction-coffee intake
*CYP3A5*	Domestication of plants; aridity (salt retention adaptation) in some Africans [60,65]	DBP-sodium intake; SBP-sodium intake; *LC*
*EDNRA*	Hypoxia - high-altitude Tibetans [66]	Arterial stiffness-physical activity
*FABP2*-rs1799883	Latitude [58]	ΔSBP-Mediterranean hypocaloric diet + exercise (MHD + E); Δglucose, fasting-MHD + E; ΔLDL-C-MHD + E; glucose tolerance-low-fat diet; glucose, fasting-low-fat diet; insulin, plasma, fasting-low-fat diet; *LC*
*FABP2*	Dispersal and subsequent exposure to novel climates; latitude [58,60]	(see above)
*GNPDA2*	Precipitation; summer [59]	Δbody weight-weight-loss intervention + exercise
*LEPR*-rs1137100	Winter PC1 [58]	SBP-physical activity; Δinsulin, fasting-physical activity
*LEPR*	Digestion of milk and dairy products; metabolism of carbohydrates, starch, proteins, lipids and phosphates; alcohol metabolism; energy metabolism, hot or cold tolerance; latitude, summer PC2; winter PC1 [58,60]	Δbody weight-low-calorie diet; insulin resistance (HOMA-IR)-PUFA, N-3, plasma; insulin resistance (HOMA-IR)-PUFA, N-6, plasma; insulin, plasma, fasting-PUFA, N-3, plasma; insulin, plasma, fasting-PUFA, N-6, plasma; ΔSBP-MHD + E; ΔBMI-MHD + E; Δbody weight-MHD + E; Δwaist circumference-MHD + E; *LC*
*LCT*	Dairy farming and milk usage; dietary preferences; alcohol consumption [60]	BMI-lactose; body weight-lactose; waist circumference-lactose
*LIPE*	Hypoxia - high-altitude Ethiopians [67]	BMI-physical activity; cholesterol:HDL-C ratio-physical activity; *LC*
*LPA*	Dispersal and subsequent exposure to novel climates; winter PC2 [58,60]	Lp (a), plasma-fish intake
*PON1*-rs662	Summer PC1 [58]	Cholesterol, total-smoking; HDL-C-physical activity; HDL-C-PUFA, N-9, oleic acid intake; LDL-C-smoking; triglyceride-smoking; *LC*
*PON1*	Dispersal and subsequent exposure to novel climates; summer PC1 [58,60]	(see above)
*PPARA*	Hypoxia - high-altitude Tibetans [62]	CRP, plasma-PUFA, N-3, percent energy from; triglyceride-PUFA, N-3, percent energy from; triglyceride-PUFA, N-6, percent energy from; triglyceride-PUFA, percent energy from; waist circumference-fat, total intake; waist circumference-SFA intake; HDL-C-PUFA intake; *LC*
*PPARD*	Dairy farming and milk usage; dietary preferences; alcohol consumption [60]	ΔHDL-C-physical activity; LDL particle size-PUFA:SFA ratio; ΔVO2max-physical activity; Δwork output, max-physical activity; *LC*
*PPARGC1A*-rs4550905	Winter PC1 [58]	BMI-alcohol consumption; BMI-physical activity; Δinsulin resistance (HOMA-IR)-low-calorie diet; *LC*
*TCF7L2*-rs11196175	Summer PC1 [58]	Glucose, fasting-fat, total, percent energy from; insulin resistance (HOMA-IR)-fat, total, percent energy from; insulin sensitivity-SFA, percent energy from; insulin, plasma, fasting-SFA, percent energy from; metabolic syndrome-SFA, percent energy from; *LC*
*UCP1*-rs1800592	Temperature - cold resistance [68]	ΔBMI-energy intake; Δbody weight-energy intake; Δwaist circumference-energy intake; *LC*
*UCP2*-rs659366	Temperature - cold resistance [68]	Δbody weight-high-calorie diet; ΔBMI-low-calorie diet; BMI-homestead; *LC*
*UCP3*-rs1800849	Temperature - cold resistance [68]	BMI-physical activity
*UCP3*	Dispersal and subsequent exposure to novel climates; winter PC1; temperature - cold resistance [58,60]	Δbody weight-high-calorie diet

When no SNP is listed, only the gene itself was shown to be under selection.

**bold** = phenotype-environment pair found in (n) studies.

*LC* = selected GxEs based less common phenotypes or environmental factors can be found in (Additional file 1).

Δ refers to a change in the phenotype, typically after an intervention.

It is a challenge to understand fully the relationships between factors driving adaptation to a given climate or geographical feature and the phenotype-environment pairings observed in published GxE interactions. Nonetheless, some examples deserve attention. GxE genes *ANGPTL4* and *PPARA*, both expressed in adipocytes, were identified as showing adaptation to high altitude in Tibetans [62,69] and as contributing to variation in HDL-C and other blood lipids (Additional file 1). Interestingly, hypoxia affects preadipocytes and adipocytes in ways that alter lipid droplet size and content, including triglyceride, and protein secretion [70,71]. The *UCP1* and *UCP2* genes are described as having undergone adaptation to temperature, specifically cold resistance [68], and participate in GxE interactions with energy intake (fuel) on BMI and body weight. Lastly, we note GxE interactions with hormone-sensitive lipase *LIPE* and physical activity. This gene resides within a region identified as having been subject to a selective sweep in Ethiopian highlanders with respect to hypoxia tolerance adaptation [67]. Overall, we believe that the observed enrichment of GxE genes for adaptation to climate and geographical traits likely originated from energy homeostasis and temperature adaptation as this dictated what food was available, how much energy was expended during daily activities, and what an individual wore (to be warm or cool). Maintaining energy homeostasis and healthy vascular function, which can be promoted by an active lifestyle, are central to diseases such as CVD, T2DM, hypertension, stroke and metabolic syndrome, which are often preceded by abnormal values of the clinical measures constituting this GxE catalog.

Although the work presented here does not explore relationships between genes under selective pressure from pathogen exposure and genes that support cardiometabolic GxE interactions [13], such instances might have relevance to the links between metabolic diseases and inflammation. In this regard, toll-like receptors, including TLR1, have roles in metabolic syndrome in macrophages and other cell types [72], and *TLR1* recently was described as having been under selective pressure in Roma gypsy and European populations in response to *Yersinia pestis*, the agent of plague [73]. *TLR4* variants support GxE interactions with obesity traits and smoking in an Argentinean population of European ancestry [74]. Identification of other immuno-metabolic genes that support cardiometabolic GxEs is intriguing but has not been explored sufficiently.

In other work, we examined our catalog of GxE variants and the GWAS-based main effect SNPs for signals of recent positive selection in populations of European ancestry with data from the 1000 Genome Selection Browser [18]. As noted in Table 2, there is no significant enrichment of positive selection based on Fst or iHS values when comparing a set of LD blocks derived from GxE interactions for cardiometabolic traits to a set of GWAS-detected LD blocks that support main effect, but non-GxE associations for the same traits. This could be interpreted in any of several ways. First, the environment indeed has exerted selective pressure on certain variants affecting cardiometabolic traits and disease risk, but the main effect GWAS associations also support as yet undescribed GxE interactions. Two, some effects of the environment are spread across the *Homo sapiens* species and are not detected as specific to populations of a single ancestry and thus may be observed as main effect associations. Three, the environmental factors driving selection at the GxE or GWAS loci could be quite different, but although these factors remain unknown, interpretation of this result is hindered. In addition, we observed no significant enrichment for Fst or iHS signals in LD blocks supporting HDL-C or physical activity GxEs compared to all cardiometabolic GxEs (data not shown). However, because a genetic marker that associates with HDL-C levels, or any other trait, may either support an as yet untested GxE interaction or a GxE for another, even unrelated phenotype, any enrichment of HDL-C GxE loci under selection compared to main effect loci cannot be fully known.

**Table 2:** Frequency of cardiometabolic GxE and GWAS SNPs under positive selection

**Dataset, all cardiometabolic traits**	**# LD blocks**	**# with Fst or iHS (%)**	***p***	**# with Fst (%)**	***p***	**# with iHS (%)**	***P***
nonGxE GWAS	549	88 (16.0)	0.61	21 (3.8)	0.66	70 (12.8)	0.44
GxE	178	30 (16.9)		8 (4.5)		22 (12.4)	

*p*-value based on two-sample *z*-test.

Seeking to add further support to the hypothesis that many environment-sensitive genes and their variants that function in human disease have been or are under selective adaptation, a theme we have explored with respect to heart disease risk [11], we examined the pathway whose genes proportionately have the greatest level of Neanderthal admixture with subsequent recent positive selection preferentially in contemporary Europeans to retain those sequences [75]. This pathway is involved in lipid catabolism and many of its 38 genes show expression divergence in brain of contemporary humans of European but not East Asian or African descent [75]. Seven of these lipid catabolism genes have been tested for GxE interactions in numerous populations: *ANGPTL3*, *APOA4*, *APOA5*, *CPT1A*, *CPT1B*, *PPARA* and *PPARD*. In non-European populations 252 different GxE tests with any of these seven genes have been performed and 33 (13.1%) were significant; in populations with European ancestry 437 such tests were performed giving 95 (21.7%) significant GxE interactions. This difference between ancestries is significant (*p* = 0.002, two-sample *z*-test) with certain implications for cardiometabolic disease risk. Furthermore, this may lend support to adaptation by Europeans to geographical specificities of that continent, but does not dismiss the possibility of complex population structure in Africa at the time of divergence of the human and Neanderthal lineages [75].

### Pathway analysis – GxE genes and cellular function

Regarding physiological and biological pathways, the phenotypes forming the GxE interactions cataloged here are generally well understood. Also, within many GxE genes there are interactions involving the same phenotype but with different environmental factors or involving the same environmental factor acting upon several phenotypes. Lastly, pathway analysis based on environmental factors, in our opinion, will be more robust once GxE GWIS results are collected and the involved variants are fully characterized. For these reasons, we opted not to perform a traditional test of pathway or gene ontology enrichment for sets of GxE genes, for example for all GxEs affecting triglycerides (TG) or all GxEs pertaining to SFA intake or even all TG-SFA GxEs, but to examine the GxE gene function in the context of metabolic syndrome (MetS). To accomplish this, we mined from a series of 12 electronic posters depicting MetS in six organs or tissues and six cell types [72] whether a gene with variants supporting a cardiometabolic GxE interaction or its encoded protein was present. We considered the presence of a gene or protein as indicative of a key function in the development or progression of MetS. Across all six cell types of adipocyte, hepatocyte, islet cell, macrophage, myocyte and neuron, we noted with interest that many GxE proteins function in a MetS context at or very near the cell surface (i.e., in the plasma membrane (PM) or physical interaction with a PM-associated protein). This is a logical site for a protein whose gene is part of an allele-specific response to an environmental stimulus, which arrives in some form at the cell surface. Similarly, it has been observed that GxE genes are enriched in cell communication and cell surface activities [76].

Second, a comparison to pathways relevant to metabolic syndrome and metabolic homeostasis [72] showed that the tissues or cell types that have the greatest frequency of genes that support GxE interactions are the adipocyte and the myocyte. From 22% to 25% of all genes depicted as pathway entities under either metabolic homeostasis or metabolic syndrome for these two cell types have evidence in the literature as participating in GxE interactions for cardiometabolic phenotypes. Other organs or cell types, such as brain (13-16%), neuron (14%), islet cell (12%), macrophage (16%) and hepatocyte (18%), have lower occurrences, a result which may arise from the high number of GxEs with physical activity. In support of these findings, a recent report on an environment-wide association study (EWAS) in the National Health and Nutrition Examination Survey (NHANES) showed that low physical activity is one of the main environmental factors contributing to all-cause mortality [77], and physical activity often lowers risk in GxE interactions. Thus, it might be more fruitful to direct efforts at identifying novel cardiometabolic GxE interactions to pathways that are functional in the adipocyte and myocyte. The other main factors contributing to all-cause mortality in the NHANES EWAS – lycopene intake, smoking status/exposure and cadmium levels – are not routinely analyzed as components of GxE interactions or high-confidence measures of intakes do not exist. When such measures are reported, genetic variation has been measured sparsely or the data are too difficult to acquire, thereby preventing thorough GxE analysis.

### GxE allele-specific effects on transcription: eQTL

We reasoned that SNPs forming GxE interactions for phenotypes that are highly relevant to a particular tissue will more frequently support allele-specific gene expression in that tissue, with a rationale similar to that showing SNPs associated with type 2 diabetes and related traits are enriched in islet cell-specific enhancers [78]. Thus, as our primary interest is in blood lipids, we examined GxEs for these traits and their relationship with expression quantitative trait loci (eQTL) in liver, as this tissue is highly relevant to these phenotypes. Of 27 triglyceride GxE SNPs, two showed eQTL in liver: rs934197 (LD with rs7575840 mapping to *APOB*) and rs1800588 (LD with rs1077834 mapping to *LIPC*). This is about a 4.9-fold (*p* <0.01, two-sample z-test) enrichment over triglyceride GxE SNPs supporting eQTL not in liver. Similarly, we found a significant enrichment of HDL-C GxE SNPs supporting liver eQTL (*p* <0.01), including rs34367192 (LD with rs10495562 mapping to *ADAM17*), rs6720173 (LD with rs3792009 mapping to *ABCG5*), and rs1800588 and rs2070895 (both in LD with rs1077834 mapping to *LIPC*). Lastly, for LDL-C traits, we observed a significant enrichment of LDL-C GxE SNPs supporting liver eQTL (*p* <0.01), including rs34367192 (LD with rs10495562 mapping to *ADAM17*), rs1800591 (LD with rs11937107 mapping to *MTTP*), and rs2070895 (LD with rs1077834 mapping to *LIPC*). All liver eQTL SNPs discussed here associate with mRNAs for the gene to which the SNP maps, except for rs7575840 associating with a transcript just upstream of *APOB*. No GxE SNPs for total cholesterol support eQTL in liver. Although the reported incidences of liver-based eQTL are small and dictate caution regarding interpretation, the consistency of the above enrichments is intriguing and suggested a comparison between GxE and GWAS signals for tissue-specific eQTL.

In order to assess the impact of the eQTL in main effects compared to environmental interactions, we tested whether CardioGxE-based LD blocks for a given trait are more likely to share a liver eQTL than GWAS-based markers. We examined LD blocks from both GxE and GWAS sources for all cardiometabolic traits and each of four main blood lipids for overlap with liver eQTL. Specifically, a comparison of GxE LD blocks and those GWAS LD blocks not overlapping with the GxE set showed no significant enrichment in liver eQTL associations, with one exception (Table 3). Notably, only one GxE LD block for total cholesterol contains a liver eQTL association and this low number gives an unreliable *p*-value of enrichment in the GWAS samples. Nonetheless, these results overall may be indicative of main effect SNPs exerting function in a tissue or cell type principal to that phenotype and the GxE SNP could be sensing differentials in environmental factors in other or peripheral tissues. Alternatively, the observation of no enrichment could indicate that there are equal effects on transcription across sources of trait variation, but these may operate in different tissues with respect to GxE and main effect. For example, brain and gut eQTL are not readily available for such analyses and GxEs may function in those organs with influences on hunger, satiety, lipid catabolism, cholesterol synthesis, or nutrient absorption. Lastly and perhaps most importantly, eQTL data are lacking for the response to a challenge that closely mimics the environmental factor in the GxE equation.

**Table 3:** Lack of enrichment for liver eQTL in GxE SNPs compared to GWAS SNPs

**Dataset**	**LD blocks: type, n**	**LD blocks with liver eQTL (%)**	***p***
All cardiometabolic traits	nonGxE GWAS, 549	35 (6.4)	0.26
	GxE, 178	9 (5.1)	
HDL-C	nonGxE GWAS, 69	8 (11.6)	0.21
	GxE, 43	3 (7.0)	
LDL-C	nonGxE GWAS, 54	6 (11.1)	0.32
	GxE, 37	3 (8.1)	
Triglyceride	nonGxE GWAS, 45	7 (15.6)	0.08
	GxE, 35	2 (5.7)	
Total cholesterol	nonGxE GWAS, 55	10 (18.2)	0.02
	GxE, 33	1 (3.0)	

*p*-value based on two-sample *z*-test.

### GxE allele-specific effects on transcription: microRNAs

Human microRNAs (miRs) have emerged as important epigenetic regulators of cardiometabolic traits [79,80]. Genetic variants involved in miR-mediated regulation have been shown to affect gene expression [81-83] and thus are suggested to contribute to phenotypic variation. As the environment can modulate miR levels, we hypothesized that GxE SNPs can function through miR-mediated regulation. In order to focus efforts on human SNPs likely to participate in miR targeting, we created a genome-wide miR regulatory SNP database (~900,000 SNPs) by integrating miR targeting prediction algorithms and databases from various resources. This comprehensive database allows assessment of the genetic effect of miR-mediated regulation on traits of interest. We searched GxE SNPs and their proxies against our miR SNP database to identify potential allele-specific miR-mRNA interactions and any miR-phenotype or miR-environmental factor relationships.

A miR SNP confidence score was created by counting for each SNP the number of supported algorithms, datasets or tables supporting a genetic effect of miR-mediated regulation in order to rank the likelihood that a SNP is a miR regulatory SNP. Confidence scores for the GxE miR SNPs and their proxies ranged from 0–13. We collected all potential (predicted and experimentally validated) regulatory miRs for each SNP with a miRSNP confidence score >3 (13 lead and 46 proxy SNPs) and identified the most frequently participating miRs among GxE miR SNPs (Table 4). Such commonly occurring miRs could serve as agents of a given phenotype or environmental factor preferentially. However, no easily discernible trends were noted, suggesting that miR-mediated regulation by GxE SNPs is highly specific or networked with other miRs. More research is needed to evaluate this. Our finding may be explained by the general understanding in the field that miR regulation is tissue specific and fine tunes gene expression in a precise physiological or metabolic response. Furthermore, as few common miRs have been assigned roles in GxE interactions or even in specific cellular challenges that imitate the environmental component of these GxEs, mechanistic interpretation of the participating alleles is difficult.

**Table 4:** Potential regulatory miRNAs involved in allele-specific miR-mRNA interactions showing GxE interactions

**SNP**	**Lead SNP**	**Gene**	**miRSNP confidence score**	Potential regulatory miRNAs*	**Cardiometabolic GxE, phenotype-environmental factor**
rs1063539	rs1063539	*ADIPOQ*	5	miR-593-3p	Obesity-PUFA, N-3, DHA + EPA, percent in erythrocyte membranes
rs12817689	rs2302706	*MMAB*	7	miR-33b-3p, miR-371a-3p, miR-371b-3p, miR-515-3p, miR-519e-3p	HDL-C-carbohydrate intake
rs1491235	rs1800591	*MTTP-TRMT10A*	5	miR-33b-5p	APOB-48 in VLDL-high-fat challenge; cholestanol/mol cholesterol, serum, fasting-diet; cholesterol in VLDL-high-fat challenge; ΔLDL-C-SFA, percent energy from; lathosterol/mol cholesterol, serum, fasting-diet; sitosterol/mol cholesterol, serum, fasting-diet
rs3734254	rs2076167	*PPARD*	11	miR-885-3p	ΔHDL-C-physical activity; Δwork output, max-physical activity
rs4225	rs5070	*APOC3*	6	miR-885-3p	HDL-C-fat, total intake; HDL-C-SFA intake
rs4707436	rs1049353	*CNR1*	12	miR-593-5p, miR-885-5p	Δcholesterol, total-MUFA, percent energy from; Δcholesterol, total-PUFA, percent energy from; ΔIL6, plasma-physical activity during energy restriction; ΔLDL-C-MUFA, percent energy from; ΔLDL-C-PUFA, percent energy from; Δleptin, plasma-physical activity during energy restriction; Δresistin, plasma-physical activity during energy restriction; ΔTNF, plasma-physical activity during energy restriction
rs4998	rs4994	*ADRB3*	6	miR-593-5p, miR-885-3p	BMI-energy intake; Δfat mass-physical activity; Δlean mass-physical activity; obesity-physical activity; triglyceride-low-calorie diet
rs5446	rs5443	*GNB3*	10	miR-33b-3p, miR-371a-3p, miR-371b-3p, miR-515-3p, miR-519e-3p	BMI-physical activity
rs7021	rs709592	*PSMD3*	9	miR-33b-3p, miR-371a-3p, miR-515-3p, miR-519e-3p	Glucose, fasting-carbohydrate, percent energy from; glucose, fasting-MUFA, percent energy from; insulin resistance (HOMA-IR)-carbohydrate, percent energy from

text should read as follows: Across all miRSNP data, the maximum confidence score was 24, and for this analysis that range was 0-13, with a higher score indicating higher confidence in the miR regulatory function.

*All miRs listed contain the prefix hsa-.

### GxE allele-specific effects on transcription: epigenetics

As DNA methylation is a well known marker for environmental change, we thought it of interest to examine whether the GxE SNPs are related to potential DNA methylation. From 180 SNPs that support GxE interactions and have unique coordinates in the dbSNP135 database, 79 (44%) either create or destroy a CpG dinucleotide, double the percent across all dbSNP135 data (22%). In addition, we find that 16 of these 79 variants map to within 3 kb of a CpG island, as downloaded from the UCSC genome browser. These results identify an accumulation of such CpG-altering SNPs (CGS), a type of SNP with particular relationships to DNA methylation [84,85], in cardiometabolic GxE interactions and suggest that these SNPs can exert impact on gene regulation in response to environmental factors and exposure over time. In this context, 5 of 16 CGSs within 3 kb of a CpG island also exhibit eQTL associations: rs659366, rs5128 (via LD to rs10047462), rs876493, rs8065443 and rs1568400. Hence, epigenetic differences that alter gene activity could underlie some inter-individual differences in obesity and other cardiometabolic phenotypes, and that relationship could be modified by both genetic and environmental factors [86]. On the other hand, of 102 human genes showing differential DNA methylation at CpG sites and differential mRNA expression of the nearest gene in pancreatic islets in a comparison of non-diabetics and T2DM subjects [87], only *ACSL5*, *IRS1* and *SLC44A4* are known to support cardiometabolic GxE interactions. That only *IRS1* participates in GxEs with glycemic phenotypes suggests a lack of evidence supporting strong connections between genetic variation, GxE interactions and epigenetics. Clearly, this analysis can be conducted more thoroughly once epigenetic and eQTL datasets expand to other tissues and cellular challenges.

### GxE allele-specific effects on transcription: gene networks and atherosclerosis

Many phenotypes discussed in the context of GxE interactions are valued by health professionals as indicators or clinical measures of risk and severity of diseases, such as stroke, myocardial infarction and type 2 diabetes. Ideally, when such a clinical indicator exceeds some threshold, a first treatment option is an adjustment to lifestyle, mainly a healthier diet and increased exercise. A recently published study identified genes expressed in mouse aorta that form the basis of the response to regression of atherosclerotic plaques independent of a different set of genes simply responding to a lowering of plasma cholesterol [88]. Although both the plasma cholesterol-lowering and plaque regression gene networks contain genes identified in GWAS for the cardiometabolic traits presented in our GxE catalog, there is a significantly higher prevalence of GxE genes over GWAS genes in these two expression networks. Of 519 GWAS genes for these traits, 80 and 174 are observed in the plasma cholesterol-lowering and plaque regression gene sets, respectively, but of 108 GxE genes associating with often measured phenotypes and environmental factors, 32 and 55 are observed in the same cholesterol-lowering and plaque regression gene sets, respectively. In both comparisons of GxE to GWAS genes, enrichment in the gene sets is significant with *p* <0.001 (Table 5). Thus, the overlap of genes responding either to plaque regression or reduction in plasma cholesterol, with genes participating in GxE interactions, of which most contain an environmental term entailing physical activity, energy from fat or total energy, is significantly more than for GWAS. This is reasonable and offers the opportunity to focus efforts to identify the genetic basis of differential responses to cholesterol-lowering dietary interventions.

**Table 5:** Plasma cholesterol-induced lesion networks are enriched for cardiometabolic GxE genes

Network, number of genes*	**Genes shared with GxE catalog**^**†**^	**Genes shared with GWAS catalog**^**‡**^	**Enrichment in GxE catalog,** ***p*-value**
Plasma cholesterol-lowering, 2697	32	80	< 0.001
Regression-reactive, 6096	55	174	< 0.001

*All genes comprising each network, as described [88].

^†^GxE genes supporting commonly measured phenotypes and involving common environmental modifiers, n =108.

^‡^GWAS genes supporting associations with cardiometabolic phenotypes, n =519.

*p*-value based on two-sample *z*-test.

## Conclusions

While it certainly may be stated that a person’s ‘genometype’ could indeed prove the most useful for individualized medicine (including individualized nutrition) and personal genetics [89], the impact of environmental interactions on a person’s panel of alleles cannot be overstated. In this regard, an interaction between an obesity genetic risk score based on 63 variants and saturated fat intake has been demonstrated in two distinct populations [90]. We have not in this analysis coalesced the genetic variants cataloged in CardioGxE around a given phenotype-environmental factor pair and processed data for a global or genometype GxE interaction, but such research could proceed with the aid of this GxE resource. Indeed, the lack of overlap between our CardioGxE dataset and published GWAS for comparable phenotypes makes evident the utility of incorporating GxEs into assessment of disease risk in two important ways. One, a GxE catalog provides the means to develop a better strategy of intervention because genetic and environmental factors combined can equip the physician for more accurate prediction of future disease risk and hence disease prevention. Two, genetic variation alone is not just diagnostic of disease risk, but is a component of and should be considered in epistatic and GxE interactions to better inform the individual of potential disease risk. Altogether, the numerous examples presented here add to the emerging view that GxEs are widespread and significant contributors to phenotypic variance [91]. Although we have highlighted instances for which more data are needed, especially taken under conditions mimicking the environmental factor of the GxE equation, the insight thus far garnered from analysis of a large GxE catalog emphasizes the influential roles of environmental factors in the genetics of complex traits, particularly those of a metabolic nature.

## Abbreviations

BMI: Body mass index; CAD: Coronary artery disease; CEU: Utah residents with ancestry from northern and western Europe, from the Centre d’Etude du Polymorphisme Humain collection; CGS: CpG-altering SNP; CVD: Cardiovascular disease; DBP: Diastolic blood pressure; eQTL: Expression quantitative trait locus; EWAS: Environment-wide association study; Fst: Fixation statistics; GWAS: Genome-wide association study; GWIS: Genome-wide interaction study; GxE: Gene by environment (interaction); HDL: High-density lipoprotein; HDL-C: High-density lipoprotein cholesterol; HOMA-IR: Homeostasis model assessment-estimated insulin resistance; iHS: Integrated haplotype score; LD: Linkage disequilibrium; LDL: Low-density lipoprotein; LDL-C: Low-density lipoprotein cholesterol; MetS: Metabolic syndrome; miR: microRNA; MUFA: Mono-unsaturated fatty acid; NHANES: National Health and Nutrition Examination Survey; PUFA: Poly-unsaturated fatty acid; SBP: Systolic blood pressure; SFA: Saturated fatty acid; SNP: Single nucleotide polymorphism; T2DM: Type 2 diabetes mellitus; TG: Triglyceride; VLDL: Very low-density lipoprotein.

## Competing interests

The authors declare that they have no competing interests.

## Authors’ contributions

LDP conceived of the study, managed the project, performed all analyses not specifically mentioned here, and wrote the manuscript. LDP and BAB mined the literature and populated the updated database. KR acquired the LD SNPs, and mined eQTL data, and LDP analyzed and interpreted those results. BEC and HSD compared GxE SNPs to GWAS databases. RH mined for cardiometabolic GxE-epistasis overlap. PDN identified GxE variants and genes under selection from climatic and geographical features. CQL identified GxE variants showing adaptation by Fst and iHS. MetS pathway analysis was undertaken by BAB. Identification of the GxE SNP effects on miR-mRNA interactions was performed by YCL. Analysis of GxE SNPs affecting potential CpG islands was done by YM. LDP and JMO have given final approval of the published version of this report, and provided financial support. All authors aided in interpretation of results, and read and revised the manuscript.

## Supplementary Material

Additional file 1 The CardioGxE catalog of gene-environment interactions for cardiometabolic phenotypes. Click here for file
